# Gender-based variations in clinical course, Endoscopic and histological findings of solitary rectal ulcer syndrome - Multicenter study from Pakistan

**DOI:** 10.12669/pjms.41.11.11998

**Published:** 2025-11

**Authors:** Nazish Butt, Ghulam Mohiuddin, Ali Akbar

**Affiliations:** 1 Nazish Butt Department of Gastroenterology, Jinnah Postgraduate Medical Centre, Karachi, Pakistan; 2 Ghulam Mohiuddin Department of Gastroenterology, Jinnah Postgraduate Medical Centre, Karachi, Pakistan; 3 Ali Akbar Department of Gastroenterology, Jinnah Postgraduate Medical Centre, Karachi, Pakistan

**Keywords:** Rectal Bleeding, Solitary Rectal Ulcer Syndrome (SRUS)

## Abstract

**Background and Objective:**

Solitary Rectal Ulcer Syndrome (SRUS) is an uncommon, chronic disorder of the pelvic floor associated with defecatory dysfunction. This prospective observational study aimed to assess the comprehensive manifestations of SRUS with a focus on sex differences in risk factors, clinical presentation, and endoscopic and histopathological findings.

**Methodology:**

The study included 132 patients (81 females, 51 males) diagnosed with SRUS at a Jinnah Postgraduate Medical Centre and National Medical Centre in Pakistan between 2017 and 2024.

**Results:**

The most commonly affected age group was 21-30 years. Clinically, rectal bleeding was the predominant symptom in both genders (74.5% males, 71.6% females; p=0.715), followed by rectal pain (41.2% males, 48.1% females; p=0.433) and altered bowel habits (15.7% males, 16.0% females; p=0.956). Self-digitation was reported by 86.2% of males and 88.8% of females (p=0.654). Endoscopically, solitary rectal ulcer was observed in 60.7% of males and 60.4% of females (p=0.973), while proctitis was present in 58.8% of males and 59.2% of females (p=0.960). Histologically, crypt distortion was noted in 43.1% of males and 29.6% of females (p=0.113), fibromuscular obliteration in 56.8% of males and 45.6% of females (p=0.211), and surface ulceration in 47.0% of males and 45.6% of females (p=0.877). Although no statistically significant gender differences were identified, the study highlights the need for larger-scale investigations to explore these patterns further.

**Conclusion:**

This study shows SRUS is more common in females 81 (61%) and commonly affects those aged 21 to 30 years, 46 (34.8%) and bleeding per rectum was the most frequent presenting symptom 96 (72.7%).

## INTRODUCTION

Solitary Rectal Ulcer Syndrome (SRUS) is a benign, troublesome, chronic disease of the pelvic floor associated with defecatory dysfunction.^1^ It is a relatively rare condition occurring in 1: 100000 (hundred thousand) individuals; however, its true incidence is masked due to overlapping clinical manifestations with Inflammatory Bowel Disease (IBD), polyps, and neoplastic conditions.^2^ The term SRUS insinuates if the lesion is single, but it is a misnomer since the lesions may be multiple and present as hyperemic mucosa and polypoidal lesion.^3^

Clinically, SRUS may present with rectal bleeding, prolonged straining, perineal and abdominal pain, a sensation of incomplete bowel evacuation, constipation, and in rare instances with rectal prolapse.^4^ Diagnosis of SRUS relies on endoscopic and histopathologic evaluation, as the condition is defined by a constellation of clinical, endoscopic, and histological changes.^5^ Endoscopic examination of SRUS may reveal a range of findings, such as single or multiple ulcers, a pseudopolypoid appearance, mid-rectal stenosis, proctitis, or nodular lesions.^2^ Histopathologically, the defining feature of SRUS is recognized as ‘fibromuscular obliteration’, often accompanied by several other abnormalities.^6^ These include surface ulceration, irregularities in glandular crypts, thickening and disorganization of the muscularis mucosa, mucous cell hyperplasia, dilated glands, and serrated or hyperplastic mucosal changes.^6^ Although the exact mechanism of SRUS is not understood properly, it is proposed that the disease results from an ischemic insult to the rectal mucosa, and as a result of vascular damage, ulcers are formed. This injury is thought to arise from direct trauma, constipation or prolonged straining and disorganized contraction of puborectalis muscle.^7^,^8^

Despite the existing literature on SRUS addressing the clinical, endoscopic, and histopathological abnormalities of the disease, there seems to be a notable gap in observing these manifestations through a gender-specific lens as management can be affected by the pregnancy and progesterone related pelvic floor changes, that may influence the treatment response. There were no studies identified that explored the potential differences in disease presentation, risk factors, or progression between males and females comprehensively. Thus, the objective of this study is to assess the comprehensive manifestations of SRUS with a focus on gender differences, aiming to explicate specific risk factors, presentation patterns, and potential implications for personalized management.

## METHODOLOGY

This prospective, observational cross-sectional study was conducted at the Gastroenterology Department at Jinnah Postgraduate Medical Centre, Karachi, and National Medical Centre, Karachi between 2017 and 2024. The diagnosis of SRUS was established when clinical presentation includes symptoms such as bleeding from the rectum, secretion of mucus, excessive straining, and sense of incomplete defecation. Endoscopic investigation revealed ulcers, inflammation, or polyp-like structures. Histological examination confirmed the diagnosis by showing fibromuscular replacement in the lamina propria, altered crypt architecture, and the presence of inflammatory cell infiltrates. Patients who turned out to have Inflammatory bowel disease and malignancy were excluded.

### Ethical consideration:

The Institutional Review Board approved the study No. F.2-81/2022-Genl/330/JPMC; Dated: December 12, 2022.

### Data collection:

Results were obtained based on questionnaires. Age, sex, height, and weight were recorded. Clinical symptoms including rectal bleeding, the use of digitation to defecate, perineal pain, mucus discharge, constipation, diarrhea and sense of incomplete evacuation, self-digitation, straining duration, rectal prolapse were recorded. Colonoscopic findings included were single or multiple ulcers, polyps, stenosis of rectum, proctitis, hemorrhoids and mass lesion and their locations were noted. Histopathological features including fibromuscular obliteration, surface ulceration, crypt distortion, glandular distortion were documented.

### Data analysis:

Data was entered and analyzed using the computer program SPSS version 26.0. Mean and standard deviation (for normally distributed data)/median and range (for skewed data) were calculated for the age. Normal data was checked using the Shapiro-Wilk test. In this study, we have compared the clinical features colonoscopic findings, risk factors, histopathology, and treatment modalities among males and females. Comparisons between groups were performed using t-tests for normally distributed variables, whereas non-normally distributed variables were analyzed by the Wilcoxon test, and the chi-squared test or Fisher’s exact test were used for categorical variables. p-values less than 0.05 were considered to be statistically significant. Furthermore, the magnitude and precision of observed differences were reported by effect sizes with 95% confidence intervals, in order to ensure that the difference is not only statistically significant but also clinically relevant.

## RESULTS

**Fig.1:**
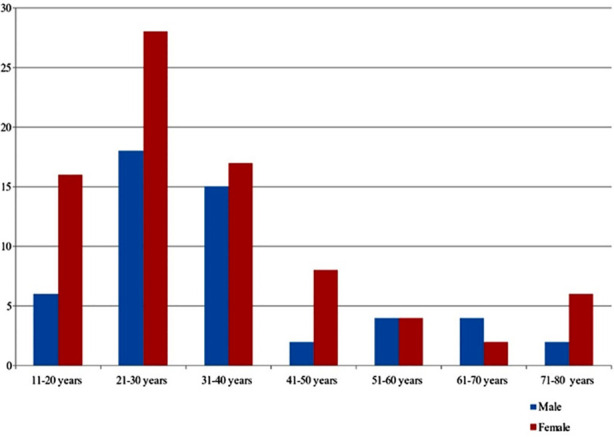
Number of male and females suffering from SRUS in different age groups.

Data from 132 patients with a confirmed diagnosis of SRUS was analyzed. The disease appeared to show a predominance towards the female gender with 81(61%) cases with 51(39%) cases in the male gender as shown in Table-I. Moreover, the most common age group associated with SRUS appears to be the age 21-30 years as shown in Fig.1. Clinically, the most common complaint was bleeding per rectum, which was observed in 74.5% males and 71.6% females (p=0.715). Rectal pain was reported by 41.2% of males and 48.1% of females (p = 0.433). Furthermore, altered bowel habits were noted in 15.7% of males and 16.0% of females (p = 0.956). Abdominal pain was more common in females (64.2%) compared to males (47.0%). Additionally, self-digitation was reported by 86.2% of males and 88.8% of females (p = 0.654). Straining during defecation was more prevalent in males (52.9%) compared to females (38.2%), with a p-value of 0.098, indicating a trend towards significance. Endoscopic findings showed solitary rectal ulcer in 60.7% of males and 60.4% of females (p = 0.973). Multiple ulcers were observed in 9.8% of males and 8.6% of females (p = 0.82). Proctitis was present in 58.8% of males and 59.2% of females (p = 0.960). According to the histological examinations, crypt distortion was noted in 43.1% of males and 29.6% of females (p = 0.113). Fibromuscular obliteration of the lamina propria was observed in 56.8% of males and 45.6% of females (p = 0.211) while surface ulceration was present in 47.0% of males and 45.6% of females (p = 0.877).

**Table-I:** Table showing gender based differences in different variables.

Variables	Male (n=51)	Female (n=81)	p-value
**Symptoms**			
Bleeding PR	38 (74.5%)	58 (71.60%)	0.715
Rectal Pain	21 (41.2%)	39 (48.1%)	0.433
Altered Bowel Habits	8 (15.7%)	13 (16.0%)	0.956
Abdominal Pain	24 (47.0%)	52 (64.2%)	0.052
Mucous	8 (15.7%)	16 (19.7%)	0.555
Tenesmus	6 (11.7%)	13 (16.0%)	0.495
Chronic Constipation	31 (60.7%)	53 (65.4%)	0.589
Obsession/ Compulsion	18 (35.2%)	33 (40.7%)	0.531
Lengthens Straining	33 (64.7%)	51 (63%)	0.839
Chronic Diarrhea	8 (15.6%)	8 (9.8%)	0.319
**Risk Factors**			
Self-Digitation	44 (86.2%)	72 (88.8%)	0.654
Straining	27 (52.9%)	31 (38.2%)	0.098
Rectal Prolapse	5 (9.8%)	6 (7.4%)	0.628
Intussipation	0 (0)	1 (1.2%)	0.426
**Endoscopic Findings**			
Solitary Ulcer	31 (60.7%)	19 (60.4%)	0.973
Multiple Ulcer	5 (9.8%)	7 (8.6%)	0.821
Proctitis	30 (58.8%)	48 (59.2%)	0.960
Mid Rectal Stenosis	2 (3.9%)	9 (11.1%)	0.146
Pseudopolypoid appearance	2 (3.9%)	6 (7.4%)	0.414
Hemorrhoids	6 (11.7%)	7 (8.6%)	0.558
Polyps	7 (13.7%)	7 (8.6%)	0.356
**Histo-pathological Findings**			
Crypts Distortion	22 (43.1%)	24 (29.6%)	0.113
Fibro muscular Obliteration of Lamina Propia	29 (56.8%)	37 (45.6%)	0.211
Extension of muscular fibers	25 (49.0%)	39 (48.1%)	0.922
Surface Ulceration	24 (47.0%)	37 (45.6%)	0.877
Gland distortion	22 (43.1%)	31 (38.2%)	0.579
**Treatment Options**			
Life style Management	47 (92.1%)	78 (96.2%)	0.301
Bio feed back	46 (90.1%)	78 (96.2%)	0.153
Surgical Referral	4 (7.8%)	3 (3.7%)	0.301

## DISCUSSION

Our study examined variations in risk factors, clinical presentation, endoscopic, and histopathological findings of SRUS with a gender-focused approach. Given the uncommon nature of the disease, there is limited data available on its various aspects. While the prevalence of SRUS in relation to gender has been explored, a significant gap remains in understanding how clinical characteristics may differ between males and females. The rarity of SRUS contributes to the scarcity and inconsistency of available literature. Study conducted by Shafiq S et al.^5^, suggest that SRUS is more common in males. In contrast, consistent with our findings, Gouriou C et al.^9^ and a network meta-analysis^10^ reported a higher prevalence among females. These discrepancies may stem from data derived from case series and smaller sample sizes. Our study, which analyzed 132 individuals, offers a relatively larger sample size given the rarity of SRUS.

The adult population is the most commonly affected by SRUS, as reported by Shahid A et al.^11^ Our study yielded similar results, with individuals aged 21–40 years being the most affected group. Interestingly, our pooled data also revealed that in population (aged 11–20 years), females were almost twice as likely to develop SRUS compared to males in the same age group. This finding contrasts with the results of Perito ER et al.^12^ These disparities may be attributed to the small sample sizes in the study as study included fewer than 15 participants. In contrast, our study had a significantly larger sample size of 132 individuals, providing more robust data.

The clinical presentation of SRUS was largely similar between males and females, with rectal bleeding being the predominant symptom observed in both genders, concordant with Gaj F et al.^10^ also reporting rectal bleeding to be the leading clinical manifestation of SRUS. The second and third most common symptoms reported by patients in our study were prolonged straining and constipation, respectively, occurring equally in both genders. The frequency of these symptoms, with respect to gender and prevalence, aligned with the findings of Derakhshani S et al.^14^ for constipation for prolonged straining. Furthermore, the most common risk factor identified in our study was the practice of self-digitation, with nearly 90% of the patients reporting this habit, distributed equally between both genders. The association between SRUS and self-digitation has also been documented by Arthi KB et al.^15^ Collectively, our findings are consistent with the most widely accepted theory regarding the etiology and pathogenesis of SRUS, which suggests that local ischemia and direct trauma are the primary causative factors. Constipation, which can result from primary issues with colonic or anorectal function or secondary causes such as organic disease, systemic conditions, or medication use, leads to increased straining during defecation. This can also often prompt patients to adopt self-digitation maneuvers, further exacerbating the problem. This behavior and the abnormal contraction of the puborectalis muscle during straining, causes local ischemia and direct trauma, ultimately resulting in rectal bleeding along with single or multiple ulcers.^16^

Given the pathophysiology and etiology of the syndrome, more than 90% of patients, regardless of gender, were treated with biofeedback therapy and lifestyle modifications. This treatment approach aligns with Ugur F.A et al.^17^ The treatment was designed to alleviate constipation as mentioned by A. Abbasi et al.^18^ By reducing straining during defecation, this approach aimed to minimize direct trauma and local ischemia.

Regarding endoscopic findings, single or multiple ulcers were equally distributed across both genders as observed by Behera MK et al.^19^ Intriguingly, proctitis, a common finding in SRUS, was slightly more prevalent in females. In addition, histopathological abnormalities, such as crypt distortion and fibromuscular obliteration, were more frequently observed in males compared to females. This observation contrasts with a study by Shafiq S et al.^5^, which reported a higher prevalence of these histological features in females.

These gender-based differences in the clinical, endoscopic, and histopathological manifestations of SRUS observed in our study, although not statistically significant, may hint at potential underlying pathophysiological mechanisms that warrant further investigation. Factors such as hormonal influences, differences in pelvic floor function, and gender-specific risk factors may contribute to these variations. Clinically, this indicates the need for a uniform approach to the management and treatment of SRUS, regardless of gender. In order to evaluate the need for a more tailored, gender-specific approach with focus on hormone analysis, a larger sample size is necessary, which could be obtained through larger, multicenter studies.

In summary, our research offers significant insights into the gender-specific clinical, endoscopic, and histopathological features of SRUS. Although no statistically significant gender differences were identified, the study underscores the necessity for larger-scale investigations to explore these patterns further. By enhancing our understanding of SRUS, our findings aim to inform future research and ultimately improve patient care.

### Strengths of the study:

One of the strengths of our study is the relatively large sample size of 132 patients, which is considerable given the rarity of SRUS. Additionally, our focus on gender differences adds a new dimension to understanding the disease’s clinical, endoscopic and histopathological characteristics. By analyzing a balanced cohort of males and females, we were able to provide more robust data regarding the gender-specific presentation of SRUS.

### Limitations

The study was conducted at only two health care center, limiting the generalisability of the findings to other populations or regions. Additionally, while our sample size is larger than previous studies, it remains relatively small for a conclusive analysis of certain variables, such as gender-specific trends in symptom presentation.

## CONCLUSION

This study shows SRUS is more common in females and commonly affects those aged 21 to 30, and rectal bleeding was the most frequent presenting symptom.

### Authors Contribution:

**NB:** Literature search, Conceived, designed the study.

**GM:** Collected data and did statistical analysis. Critical Review

**AA:** Has written and edited the manuscript, reviewed and final approval of manuscript and is responsible for integrity of research.

All authors have read and approved the final manuscript.
